# Study protocol: A stepped-wedge, cluster-randomized trial of the effectiveness of a deliberative loop in identifying implementation strategies for the adoption of a dental sealant guideline in dental clinics

**DOI:** 10.1186/s43058-021-00199-6

**Published:** 2021-08-28

**Authors:** Deborah E. Polk, Erick G. Guerrero, Inga Gruß, Nilesh H. Shah, Nadia M. Yosuf, Tim Dawson, Charles D. Kaplan, Daniel J. Pihlstrom, Jeffrey L. Fellows

**Affiliations:** 1 University of Pittsburgh School of Dental Medicine, Pittsburgh, PA USA; 2 Research to End Healthcare Disparities Corp, Los Angeles, CA USA; 3 Kaiser Permanente Center for Health Research, Portland, OR USA; 4 The Art of Democracy, Pittsburgh, PA USA; 5 Charles D. Kaplan Consulting, LLC, Los Angeles, USA; 6 Permanente Dental Associates, Portland, OR USA

**Keywords:** Implementation strategy, Oral health, Dental caries, Dental sealants, Study protocol

## Abstract

**Background:**

The American Dental Association (ADA) recommends dental providers apply dental sealants to the occlusal surfaces of permanent molars for the prevention or treatment of non-cavitated dental caries. Despite the evidence-based support for this guideline, adherence among general dentists is low, ranging from less than 5 to 38.5%. Thus, an evidence-to-practice gap exists, and it is unclear which implementation strategies would best support providers in adopting and implementing the evidence-based practice. One potential approach to selecting and tailoring implementation strategies is a deliberative loop process, a stakeholder-engaged approach to decision-making. This trial aims to test the acceptability, feasibility, and effectiveness of using a deliberative loop intervention with stakeholders (i.e., providers and staff) to enable managers to select implementation strategies that facilitate the adoption of an evidence-based dental practice.

**Methods:**

Sixteen dental clinics within Kaiser Permanente Northwest Dental will be cluster randomized to determine the timing of receiving the intervention in this stepped-wedge trial. In the three-part deliberative loop intervention, clinic stakeholders engage in the following activities: (1) receive background information, (2) participate in facilitated small-group discussions designed to promote learning from each other’s lived experiences and develop informed opinions about effective clinic-level implementation strategies, and (3) share their informed opinions with clinic leaders, who may then choose to select and deploy implementation strategies based on the stakeholders’ informed opinions. The primary outcome of Reach will be defined as patient-level receipt of guideline-concordant care. Secondary outcomes will include the cost-effectiveness, acceptability, and feasibility of the deliberative loop process. Implementation strategies deployed will be catalogued over time.

**Discussion:**

These results will establish the extent to which the deliberative loop process can help leaders select and tailor implementation strategies with the goal of improving guideline-concordant dental care.

**Trial registration:**

This project is registered at ClinicalTrials.gov with ID NCT04682730. The trial was first registered on 12/18/2020. https://clinicaltrials.gov/ct2/show/NCT04682730

Contributions to the literature

- Little guidance currently exists for how organizations should select implementation strategies.
- The purpose of this study is to test whether the deliberative loop process helps an organization select implementation strategies.
- The results of this study will be of interest to any organization considering how best to select implementation strategies to deploy and to the field of implementation science as a whole.

## Background

Non-cavitated occlusal carious lesions (NCCLs), also known as incipient caries or white spot lesions, are lesions in which bacteria have compromised some but not all of the enamel (i.e., outermost layer) on the occlusal surface of the tooth. If left untreated, the bacteria may continue to harm the tooth structure and the lesion may continue to progress to the point at which a filling is needed. Receiving a filling involves having healthy tooth structure removed, which increases the risk of root infection, tooth fracture, and tooth loss, and the financial cost of managing these consequences [1, 2].

The most effective approach to both preventing and arresting the progression of NCCLs is dental sealants [3]. The effectiveness of sealants far exceeds the benefits provided by commonly used interventions such as topical fluoride applications and professional dental cleanings [3]. Despite the evidence base supporting and American Dental Association (ADA) guidelines recommending the use of dental sealants [4], provider compliance with the guideline remains low. Among general dentists, guideline compliance ranges from less than 5 to 38.5% [5, 6]. In a descriptive study of the dental clinics participating in the present intervention, although almost 95% of the general and pediatric dentists reported using sealants to prevent caries, only 51% reported using sealants to arrest NCCLs [7]. Furthermore, of the approaches endorsed, using sealants to arrest NCCLs was the approach least frequently adopted. Dentists reported using less effective approaches such as providing office remineralization (67%), prescribing a behavioral intervention (66%), indicating a “watch” in the chart (58%), and placing a restoration (58%). Consistent adherence to the guideline will improve NCCL treatment outcomes and may lead to reduced treatment cost.

The deliberative loop process is a novel implementation intervention that may be well-suited to helping large group dental practices select and tailor implementation strategies to address implementation barriers. Implementation strategies are “methods or techniques used to enhance adoption, implementation, and sustainability of a clinical program or practice” [8]. Selecting and tailoring implementation strategies is a challenge that is being addressed in several ways by implementation scientists [9]. However, to date, the deliberative loop process has not been studied as a method to assist leaders in selecting and tailoring strategies. The deliberative loop process is a three-step approach entailing educating stakeholders, engaging stakeholders in facilitated conversations about strategies, and informing leaders about strategies stakeholders recommend [10]. This approach is both promising and unique because it draws from the collective wisdom of those affected by the policy and engages differences in perspective as a resource.

The objective of the Dissemination and Implementation of Sealant Guidelines in Organizations (DISGO) study is to determine whether introducing the deliberative loop process to an organization enables the organization to improve adherence to a guideline based on the ADA’s pit-and-fissure dental sealant guideline. This protocol paper outlines the methods for a trial evaluating whether the deliberative loop process increases guideline-concordant care. We hypothesize that introducing the deliberative loop process will enable providers and staff to develop informed understandings of the barriers to implementing the guideline, identify potential implementation strategies to overcome the barriers, and share their opinions with leadership. Using the feedback from providers (dentists, dental hygienists, and expanded function dental assistants) and staff, leadership (decision makers including upper and middle managers) will be able to identify and adopt implementation strategies, which will lead to providers placing more sealants. In addition, we are evaluating the cost-effectiveness of the deliberative loop intervention.

## Methods

### Conceptual model

**Fig. 1:**
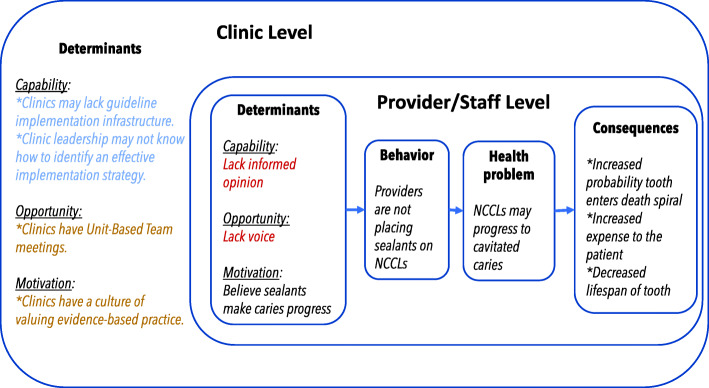
Conceptual model. *Note*. Blue denotes barriers targeted by the deliberative process intervention, gold denotes facilitators, and red denotes mechanisms proposed to be influenced by the intervention

Figure 1 illustrates our conceptual model, derived from our formative work with stakeholders to identify barriers and facilitators [11]. The determinants are framed using the COM-B model of behavior [12], which posits that an agent’s behavior changes when that agent has sufficient capability, opportunity, and motivation to change. We found that at the clinic level, the main barrier is one of capability. The organization’s current implementation strategy relies on the passive dissemination of guidelines through formal and informal staff meetings, continuing education, and clinic champions. At the provider and staff level, the main capability barrier is that individual providers and staff members lack a complete understanding of the barriers to implementing the guideline, and thus, they may be unable to recommend potential implementation strategies to overcome the barriers (i.e., informed opinion). Providers’ and staff’s main opportunity barrier is that they may lack the opportunity to share informed recommendations for implementation interventions with leadership (i.e., voice). An important motivational barrier for many dentists is the conflict in clinical decision making between dentist autonomy and evidence-based practice. In addition to barriers, we also identified facilitators. At the clinic level, the main opportunity facilitator is the existence of the Unit-Based Team meeting, in which all clinic providers and staff come together to develop solutions to problems. The main motivation facilitator is the practice’s commitment to evidence-based practice.

### Study setting

The Kaiser Permanente Dental (KPD) program is part of the Kaiser Permanente Northwest (KPNW) integrated health care system and provides comprehensive, pre-paid dental care services to over 250,000 dental plan members in Oregon and southwest Washington. The organization employs 635 administrative and clinical support staff across its 21 dental offices. With the exception of administrative and managerial staff, all front-line staff are unionized. Permanente Dental Associates (PDA) is a professional corporation of 150 associate and shareholder dentists and denturists that provides comprehensive care to KPD members. KPD insurance plans offer an array of different insurance benefits to employers and individuals. Across all KPD plans, there are no age or frequency limitations for dental sealant placement, which reduces the financial barrier for patients when a dental sealant is indicated. In addition, KPD is part of Health Share of Oregon (www.healthshareoregon.org), a large coordinated care organization that services the state’s Medicaid population in the tri-county Portland metropolitan area.

Kaiser Permanente has a long history of engaging with unionized workforces. The organization uses a labor management partnership in many of its operational initiatives. At the level of the dental clinic, this partnership is manifested in a self-directed decision-making structure, called the Unit-Based Team (UBT), and is co-led by a manager, a staff member, and a dentist. The team includes all the members of the natural work group, which enables them to build on the diverse perspectives and expertise of the team members. The purpose of the UBT is to address issues affecting performance or the work environment. For example, if a clinic wanted to increase patient satisfaction, the team might decide to provide patients with spa-like amenities, such as eye masks and heated blankets. The UBT meetings are clinic-based and occur monthly, typically during a lunch hour. All employees present at a clinic on that day, including front-of-the-house staff, attend and participate. If information directly related to patient care or employment is discussed at the UBT meeting, the clinic manager may send that information to employees who were not present. The organizational structure provides substantial clinic-level autonomy to organize care to fit different patient panels and office practice styles.

### Study design and recruitment

**Fig. 2:**
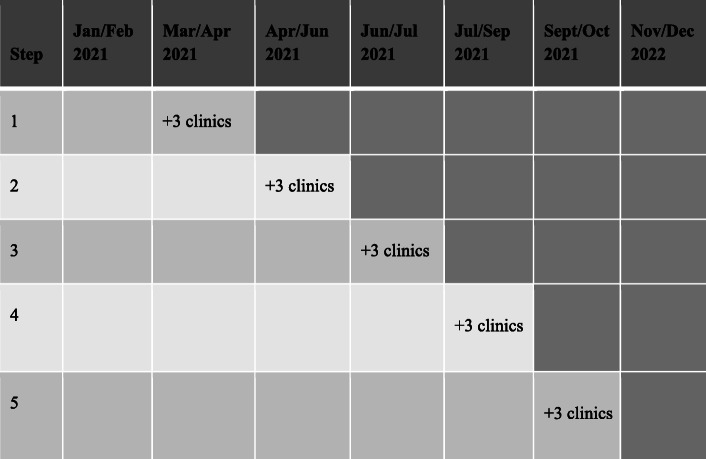
Schedule of exposure of clinics by cluster to the intervention

**Fig. 3:**
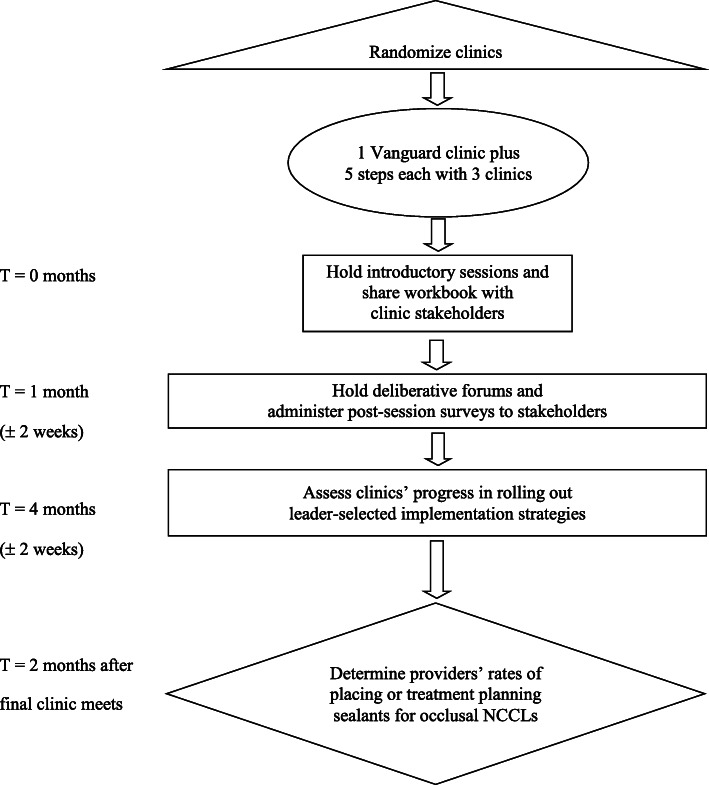
CONSORT flow diagram

The DISGO study uses a cluster-randomized, stepped-wedge design to evaluate the effectiveness and cost-effectiveness of the deliberative loop intervention compared with a non-intervention period. To form the clusters, we used clinic volume (annual visits) and provider (general and pediatric dentists and dental hygienists) staffing FTEs during 2019 (before COVID-19) to categorize 16 of 21 dental offices by size as small, medium, or large. We excluded five offices that were reorganized in 2020 to focus on urgent care. To compose a cluster, we randomly selected one clinic from each of the three size categories. One remaining medium sized clinic serves as the vanguard clinic. The intervention was launched in the vanguard clinic in March 2021 and was followed by the first cluster in April. Clusters will roll out in five 6-week periods, for a total of 30 weeks. This rollout schedule will provide 98 non-intervention months of data and 101 intervention months (Table 1) for evaluation during a 12-month evaluation period from January 1 to December 31, 2021 (Figs. 2 and 3). The study adheres to the SPIRIT guideline [13, 14].

**Table 1:** Distribution of clinic-months over the study period

		Number of clinics	
Month	Number of clinics receiving intervention	Non-intervention period	Intervention period
Jan		16	0
Feb		16	0
Mar	1	15	1
Apr	1	14	2
May	2	12	4
June	3	9	8
July	1	8	10
Aug	2	6	13
Sept	4	2	15
Oct	2	0	16
Nov		0	16
Dec		0	16
Total	16	114	117

*Note*: If the clinic’s deliberative forum occurs with the first 15 days of the month, the intervention period is counted as starting in that month; if the clinic’s deliberative forum occurs within the last 15–16 days of the month, the intervention period is counted as starting in the month following

All persons employed by KPD or PDA and who work in the 16 randomized Kaiser Permanente dental clinics, part- and full-time, will be eligible to participate in the study. This includes both service providers (dentists, dental hygienists, and expanded function dentist assistants) and front-of-the-house staff. No patients are participating in this study.

### Power

Power was calculated using the “stepped wedge” package in Stata 16 [15], based on the approach developed by Hussey and Hughes [16]. Our null hypothesis is that providers’ rates of placing or treatment planning sealants for occlusal NCCLs will not differ between the non-intervention and intervention periods. Our alternative hypothesis is that providers’ rates of placing or treatment planning sealants for occlusal NCCLs will increase following the intervention. We assumed a Type I error of 0.05 and a 3% rate of placing or treatment planning sealants in the non-intervention period and a 10% rate in the intervention period, which is an increase that is similar to that seen in similar interventions [17]. Scenarios were calculated using intraclass correlations of 0.01, 0.1, and 0.25 and *n*=50 as the average number of providers per clinic in a conservative scenario and *n*=60 in a more realistic scenario using provider counts from participating clinics. Randomizing 15 clinics along with 1 vanguard clinic using a stepped-wedge approach with these assumptions and under these scenarios, we will have at least 80% power and close to 100%.

### The deliberative loop intervention

**Fig. 4:**
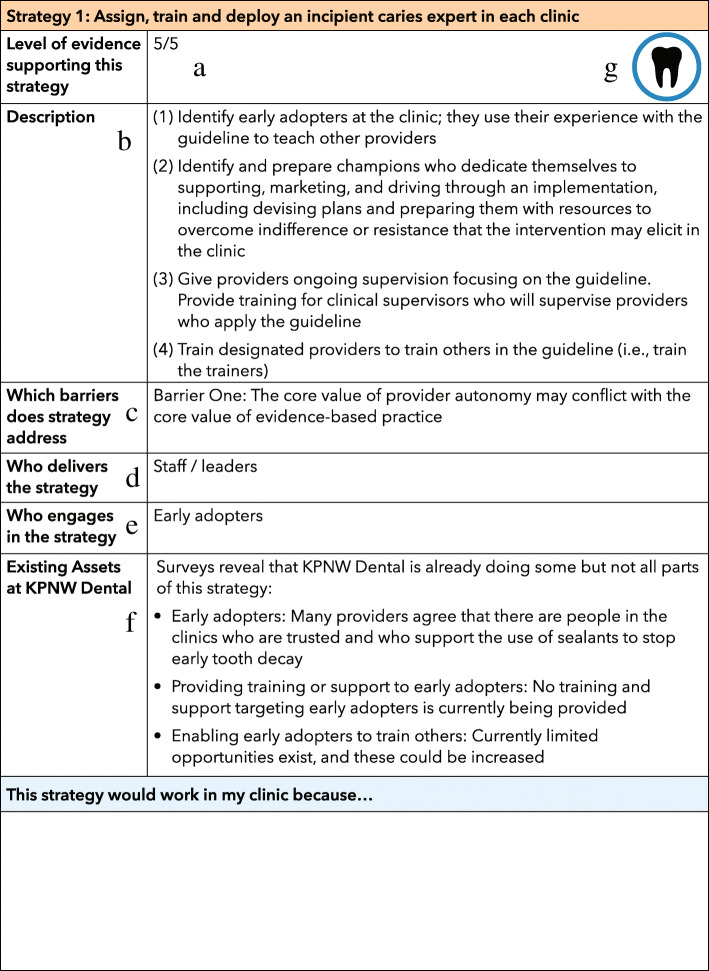
Example of information provided in the workbook for one implementation strategy. *Note*. (a) A rating of the level of evidence supporting the strategy. (b) A brief description of the strategy. (c) An anchor to the barrier(s) the strategy could address. (d)Who delivers the strategy. (e) Who engages in the strategy. (f) A summary of existing assets at KPD relevant to the strategy. (g) A unique badge for the strategy

The deliberative loop intervention is designed to address barriers while building on facilitators. The clinic-level implementation intervention is to introduce to the clinics the deliberative loop process for identifying clinic- and provider-level implementation strategies. There are three steps to a deliberative loop. In the first step, during an introduction to the process presented via a prerecorded PowerPoint presentation delivered over Microsoft Teams, stakeholders (i.e., providers and staff) receive background information designed to include the full range of perspectives on the issue. They also receive a workbook via email with information about barriers and possible implementation strategies (Fig. 4).

In the second step, about a month after receiving the background information, the stakeholders participate in an online facilitated small group discussion (i.e., deliberative forum) using the Common Ground for Action platform (Kettering Foundation and the National Issues Forums Institute, Dayton, Ohio), in which they share their lived experience and hear the lived experience of their fellow stakeholders. Groups are designed to include stakeholders representing the range of roles in the clinic and thus perspectives on the issue being addressed. Achieving consensus is not a goal of the process; rather, the goal is to expand stakeholders’ understanding of the issue.

In the third step, immediately following the deliberative forum, the stakeholders complete a survey accessed via Qualtrics (Seattle, Washington and Provo, Utah) sharing their now-informed opinion about the implementation strategies they recommend. Two weeks following the deliberative forum, the results of the survey are summarized in a report and shared with leadership, who selects the implementation strategy. Thus, the product that results from the deliberative loop process is a set of recommendations from stakeholders for clinic- and provider-level implementation strategies for leadership to consider. Due to COVID-19 and the restrictions around in-person meetings, all meetings are conducted online from workstations in the dental offices.

Using the Theoretical Domains Framework [18], the deliberative loop intervention engages the following intervention functions (see Table 2): environmental restructuring, education, and training [18, 19]. The specific interventions targeting these functions include restructuring the social environment, instruction on how to perform the behavior, behavioral practice/rehearsal, and behavioral experiments [19]. The environmental restructuring comprises the introduction of the deliberative loop process into the organization’s approach to implementation. Instruction on how to perform the behavior occurs when the stakeholders learn about the deliberative loop process during the introductory session. The behavioral practice/rehearsal occurs when the stakeholders experience the three steps of the deliberative loop process. And the behavioral experiment occurs when they apply the deliberative loop process to the problem of low adherence to the pit-and-fissure dental sealant guideline. Because the implementation strategy for guideline adherence will be identified by leadership following the deliberative loop process, we are unable to categorize the strategy.

**Table 2:** Relationships among COM-B constructs, barriers, facilitators, intervention functions, behavior change techniques, and modes of delivery

COM-B Construct	Targeted barrier	Facilitator	Intervention function	Behavior change technique	Mode of delivery
Capability	Lack of knowledge of how to identify implementation strategies		Education	Instruction	Introductory session
			Training	Behavioral practice/rehearsal	Practice with deliberative loop
				Behavioral experiment	Application of the deliberative loop process to the lack of adherence to the guideline
	Lack of decision-making process		Environmental restructuring	Restructuring the social environment	Deploying the deliberative loop process in the clinics
Opportunity		UBT meeting			
Motivation		Value evidence-based practice			

### Measures

Table 3 outlines the measures, sources, and timing for each construct in the conceptual model and study.

**Table 3:** Theoretical constructs, measures, data sources, and timing of data collection

Theoretical construct	Measure	Data source	Timing of data collection
Primary outcome: Provider sealing behavior	*Percentage of eligible lesions receiving a sealant	Electronic health record	B, E
Secondary outcome: Practice cost outcomes	*Total cost, including total program costs, total costs per clinic, and total costs per member per month *Incremental cost effectiveness ratio	Clinic managers and other KPD and PDA staff	B, E
Adoption	*Percentage of introductory sessions, deliberative forums delivered *Percentage of stakeholders attending introductory sessions and deliberative forums *Percentage of post-session surveys completed	Tracking logs, Provider/staff survey	I
Fidelity	*Fidelity of deliberative forums *Percentage of reports delivered to leadership	Tracking logs, Provider/staff survey, transcript coding	I
Acceptability	*Leadership and provider/staff ratings of acceptability of the deliberative loop process	Leadership ratings, Provider/staff survey	I, E
Mechanism: Informed opinions	*Change in top five strategies from the start to the end of the deliberative forum	Output from Common Ground for Action platform	I
Mechanism: Voice	*Difference in Promotive & Prohibitive Voice between UBT meeting and deliberative forum *Perception that leadership will take opinion into consideration *Qualitative analysis of transcripts	Provider/staff survey, transcript coding	I
Implementation strategies selected and adopted	*Percentage of clinics selecting an implementation strategy *Percentage of clinics deploying the selected implementation strategy	Tracking log Provider/staff interview	Q

*Note*. *B* baseline, *I* intervention, *Q* three months after deliberative forum, *E* end of study

#### Primary outcome: provider sealing behavior

The primary outcome, a measure of Reach, the extent to which a program attracts its intended audience [20], will be calculated for each clinic and for each dentist as the difference in the rates of sealant application for occlusal E1/E2 lesions between the intervention and non-intervention periods. This represents patient-level receipt of guideline-concordant care. Providers’ rates of sealant application for occlusal E1/E2 lesions will be extracted from the electronic health record.

#### Secondary outcome: cost and cost-effectiveness

The secondary outcome, a measure of implementation, is the cost and cost-effectiveness of the deliberative loop intervention. We will estimate total program costs, total costs per clinic, and total costs per member per month (PMPM) for the non-intervention and intervention periods. We will assess the cost-effectiveness of the deliberative loop intervention by estimating an incremental cost-effectiveness ratio (ICER) to quantify the additional intervention costs associated with increasing the sealant placement rates during the intervention period compared with usual care during the non-intervention period.

#### Adoption

Project staff will keep records of the number of introductory sessions and deliberative forums delivered to the clinics and the percentage of expected stakeholders who attended the sessions. Project staff will count the number of surveys submitted and compare the count against the clinic census provided by the Clinic Manager.

#### Fidelity

Because the introductory session is delivered via a prerecorded video, the workbook is sent to all clinic staff via email, and the link to the post-session survey is embedded in the deliberative forum online platform, assessment of fidelity of the deliberative loop intervention will focus on the deliberative forum, which will be evaluated in two ways, and the report provided to leadership.

First, all deliberative forums will generate a written transcript. Following each deliberative forum, we will review the transcripts using four codes to assess how well the facilitator adhered to the protocol. An example of one of the codes is as follows: “Elicits viewpoints from every participant/ensures that no one dominates the discussion: Encourages equal participation of each participant, makes room for participants to share their opinions.” In addition, we will review the transcripts using two codes to assess whether the stakeholders participated in the facilitated discussions. An example of one of the codes is as follows: “Unique contributions that include statement of opinion, statement of reason.”

Second, included in the post-session survey are two items assessing fidelity (e.g., “Today’s discussion caused me to consider points of view that I had not previously considered”) scored on a 5-point Likert-type scale ranging from “Strongly Disagree” to “Strongly Agree” together with a free response box for comments and suggestions. If we determine from the fidelity assessment that the training for the facilitators could be improved, we will change their training as indicated.

To determine whether reports summarizing the stakeholders’ informed opinions are shared with leadership, project staff will track the percentage of reports the study team delivers to leadership.

#### Acceptability

We will assess the acceptability of the intervention to both leadership and stakeholders. Following the final deliberative forum, we will assess KPD’s and PDA leadership’s perceptions of acceptability and feasibility of the deliberative loop process. We will share results from our cost and cost-effectiveness analyses with the clinic, KPD, and PDA management teams and have them rate the acceptability and feasibility of the deliberative loop on 5-point Likert scales.

Immediately following each clinic’s deliberative forum on the post-session survey, we will obtain from stakeholders measures of the acceptability of the deliberative forum and a regular UBT meeting. Stakeholders will rate all questions on a 5-point Likert-type scale ranging from “Strongly Disagree” to “Strongly Agree” and may add comments and suggestions in a free response box. We will assess the acceptability of the workbook providing background information via three items (e.g., “I found the written materials clear and easy to understand”). We will assess the helpfulness (three items; e.g., “I found the group discussions informative”) and relevance (five items; e.g., “Today’s discussion would be a good way for us to explore other issues”) of the group discussions. We will assess stakeholders’ comfort using the online platform.

#### Mechanisms

We propose that the deliberative loop intervention will change stakeholder’ opinions and their perceptions of voice. To measure change in stakeholders’ opinions, we will quantify the absolute change in stakeholders’ rankings of their top five strategies (out of a total of eight possible) made at the beginning and end of the deliberative forum. For options that are not ranked at either the beginning or end of the forum, we will assign them a value of 6. Thus, for example, if an option was not ranked at the beginning of the forum but then ranked 3 at the end of the forum, we will calculate the change as 3.

We will measure stakeholders’ perceptions of voice, which we define as having the opportunity to share one’s views, in three ways. First, voice will be measured using the Promotive and Prohibitive Voice scales [21]. Both Promotive Voice and Prohibitive Voice will be assessed immediately following the deliberative forum, via the post-session survey. Stakeholders will complete the scales first as they characterize the deliberative forum and second as they characterize past UBT meetings. Each scale has five items and is scored on a 5-point Likert-type scale ranging from “Strongly Disagree” to “Strongly Agree.” Six-week test-retest reliability has been shown to be 0.42 for Promotive Voice and 0.44 for Prohibitive Voice. In validation studies, these scales have been shown to have convergent and discriminant validity. Confirmatory analysis that also included measures of psychological safety, felt obligation for constructive change, and organization-based self-esteem demonstrated that a five-factor model in which each scale loaded on a separate factor fit best.

Second, the post-session survey will also include questions addressing the stakeholders’ perceptions of leadership responsiveness to feedback in the past year (three questions) and anticipated responsiveness to the feedback from the forum. These questions are scored on a 5-point Likert-type scale ranging from “Strongly Disagree” to “Strongly Agree” and are followed by a free response box for comments and suggestions.

Third, we will review the transcripts from the deliberative forums using six codes to assess stakeholder voice. An example of one of the codes is as follows: “Suggest new behaviors which are beneficial to my clinic.”

#### Exploratory measures

To learn more about any effects of the deliberative loop process, project staff will track the percentage of clinics selecting an implementation strategy and deploying their selected implementation strategy. Either the Clinic Manager or a champion identified by the Clinic Manager will track any strategies implemented using a tracking sheet designed for the study and will share the tracking sheet with the study team quarterly. In addition, 3 months after a clinic’s deliberative forum, the Clinic Manager will identify 1–2 clinic staff to participate in a semi-structured interview. The tracking sheets will serve as the basis for the interview, which is designed to obtain additional information about the implementation process. An example of one of the interview questions is as follows: “To your knowledge, was there any follow-up from leadership about possible implementation of strategies to improve sealant placement rates?”

### Data analysis

#### Primary outcome analysis

The non- and intervention sealant rates and their difference will be treated as continuous variables. Using an intention to treat approach, we will use multi-level modeling to compare the sealant rates between the intervention and non-intervention periods. We will use generalized linear models or generalized estimating equations to model the intervention effect while nesting sealant outcomes within provider and provider within clinic and accounting for secular trends, if necessary. In this scenario, our outcome will be whether an eligible E1/E2 lesion was sealed, and we will examine the clinic-level effects of the intervention [22].

The PDA guideline includes children, adolescents, and adults. To enable comparison with studies following the ADA guideline, in addition to analyzing lesions occurring in children, adolescents, and adults, we will also conduct analyses for lesions occurring in just children and adolescents. Per the ADA, we will define “children and adolescents” as persons ranging in age from 6 through 17 years old.

#### Secondary outcome analysis

We will use Program Cost Analysis [23] to quantify total program costs, total costs per clinic, and total costs per member per month (PMPM) for the non-intervention and intervention periods. We will work with KPD and PDA staff to develop realistic real-world estimates of the staff time and resources used to develop and print a workbook, including staff time that would be needed to conduct an appropriate literature review, collect and assess health records data, develop clinic monitoring processes, and prepare, print, and distribute a completed workbook. Staff time for meetings, workbook review, the deliberative forum, post-forum survey, and relevant clinic follow-up meetings will be documented, and average labor costs, by profession type, will be assigned. Costs for selected implementation strategies (equipment, trainings, CE, staff time) will be obtained from clinic managers and other KPD and PDA staff during the implementation period. Treatment costs (procedure costs based on local market paid claims) for eligible tooth surfaces and patient-paid costs will be obtained from the electronic health records. All costs will be reported in 2021 dollars.

An incremental cost-effectiveness ratio (ICER) will be estimated using the following formula: (mTCi − mTCc) / ( mSPRi − mSPRc), where mTCi and mTCc represent the mean total intervention costs for clinics during the non-intervention and intervention periods, including NCCL treatment costs, and mSPRi and mSPRc represent the mean cumulative sealant placement rates for eligible teeth among patients with a routine care visit over the 12-month evaluation period for each clinic. We will calculate ICERs and bootstrapped confidence intervals methods based on the data [24]. We expect differences in both costs and sealant placement rates to be unequivocally positive. If, however, the results are equivocal, we will conduct simulation modeling using bootstrapping and incremental net benefit approaches to assess the effects of model input variability on outcomes [25–27]. Incremental net benefit analysis allows adjustment for known statistical issues associated with confidence intervals (CIs) for equivocal ICERs by estimating CIs around the organizations (payer’s) willingness to pay for the intervention.

#### Exploratory analysis of putative mediators

We will examine whether having an informed opinion and perceptions of voice mediate the relationship between exposure to the intervention and the use of sealants on NCCL. We will use causal inference methods [28] that have been extended to allow for multiple mediators, as well as exposure-mediator and mediator-mediator interactions. This framework includes both regression-based approaches and approaches based on inverse probability weighting. We will default to the regression-based approach. If model fitting issues are present, we will use the inverse probability weighting approach.

#### Additional analyses

We will use descriptive analyses to characterize adoption, fidelity, and acceptability. In addition, we will use descriptive statistics to characterize clinics’ selection and deployment of implementation strategies.

## Discussion

The purpose of this study is to examine whether introducing the deliberative loop process to an organization enables the clinics within the organization to identify implementation strategies for the organization’s pit-and-fissure dental sealant guideline. The deliberative loop process is a decision-making process that draws on the lived experience of stakeholders and engages difference as a resource. This study will determine whether the deliberative loop process assists clinic leadership at an organization that values and practices shared decision-making in selecting implementation strategies that improve provider adherence to a guideline. There is little information about effective implementation processes for large group dental practices. Our study may be the first to introduce a process that includes both top-down and bottom-up elements. The top-down element supports clinic leadership in their decision-making powers. The bottom-up element elicits the desirability to providers and staff of a range of clinic-level implementation interventions and provides this information to clinic leadership.

Strengths of the study include being guided by theory, having an intervention that addresses identified barriers, and receiving strong support from KPD leadership. With this support, we were able to randomize clinics to steps and optimize the intervention to the needs of the clinics. There are limitations that must be considered, as well. The intervention comprises three components. Because all clinics are receiving all components, should the intervention be successful, we will not have information about the components that contribute to that success. Additionally, because we are conducting this study in the context of one organization with its culture of shared decision-making, should the intervention be successful, we will not be able to determine the cultural parameters that contribute to that success. Finally, the measures characterizing perceptions of voice during the deliberative forum and UBT meetings are both taken following the deliberative forum. Although the retrospective measure of voice during UBT meetings could lack validity and thus bias the results, we believe that the measure could be more valid because the stakeholders have an alternative, the deliberative forum, to use as a comparison. This study will not be able to assess this possibility, however.

## Conclusion

One aspect of an implementation process involves selecting the implementation strategy to deploy. This study seeks to evaluate the effectiveness of the deliberative loop process, in which leaders select implementation strategies (i.e., top down) informed by the opinions of those who will be affected by the policy (i.e., bottom up), to increase provider adherence to the pit-and-fissure dental sealant guideline. The results of this study will be of interest to any organization considering how best to select implementation strategies to deploy and to the field of implementation science as a whole.

## Data Availability

Data sharing is not applicable to this article as no datasets were generated or analyzed during the current study.

## Abbreviations

ADA: American Dental Association
CEA: Cost-effectiveness analysis
CI: Confidence interval
DISGO: Dissemination and Implementation of Sealant Guidelines in Organizations
E1/E2: Enamel lesions
EHR: Electronic health record
ICER: Incremental cost-effectiveness ratio
KPD: Kaiser Permanente Dental
mSPRi: Mean cumulative sealant placement rates for eligible teeth among patients with a routine care visit at clinics following the intervention
mSPRc: Mean cumulative sealant placement rates for eligible teeth among patients with a routine care visit at clinics before the intervention
mTCi: Mean total intervention costs at clinics following the intervention
mTCc: Mean total intervention costs at clinics before the intervention
NCCL: Non-cavitated carious lesion
PDA: Permanente Dental Associates
PMPM: Per member per month
UBT: Unit-Based Team
